# Supplementary figures and images for: Combination of JAKi and HDACi Exerts Antiangiogenic Potential in Cutaneous T-Cell Lymphoma
Source: Cancers (Basel). 2024 Sep 17;16(18):3176. doi: 10.3390/cancers16183176 (PMC11430229; doi:10.3390/cancers16183176)

**Supp**

**Myla**

**SeAx**

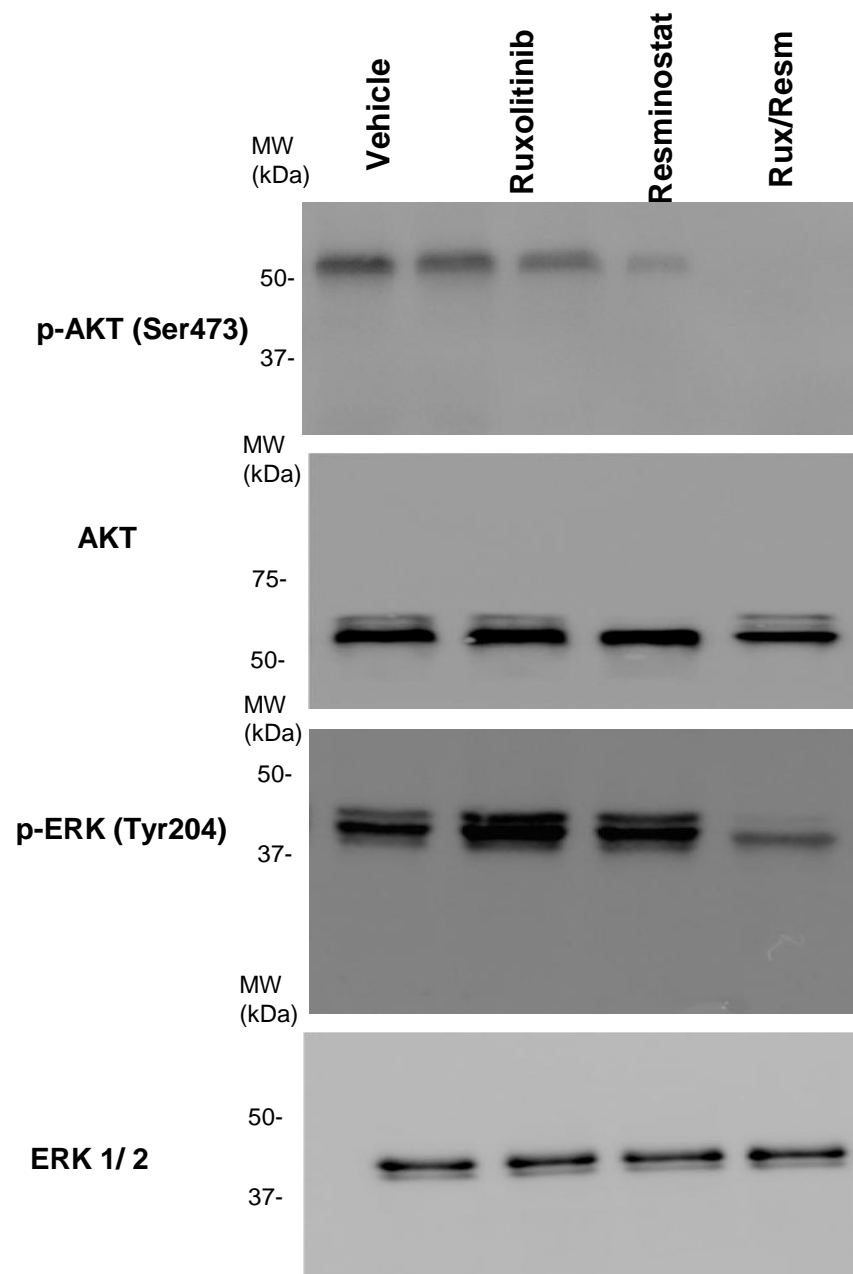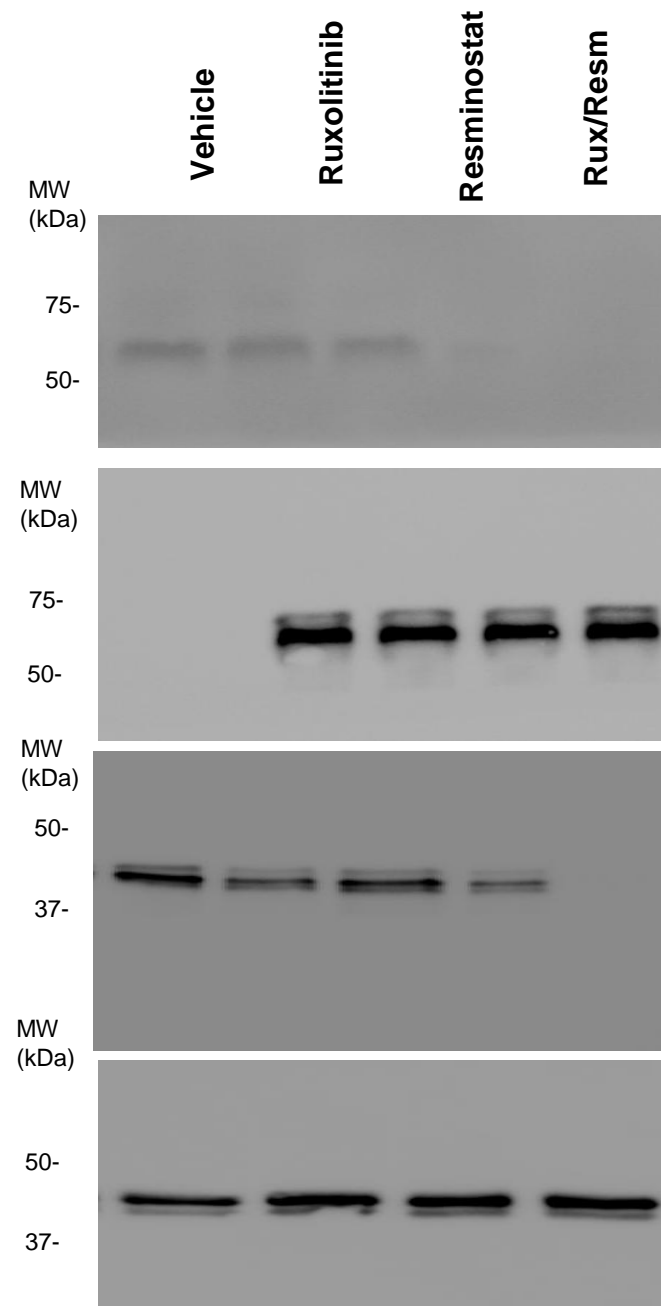

Supplement: Supplementary file 1 [file cancers-16-03176-s001.zip › cancers-3169622-supplementary.pdf]
